# Effect of a Popular Web Drama Video Series on HIV and Other Sexually Transmitted Infection Testing Among Gay, Bisexual, and Other Men Who Have Sex With Men in Singapore: Community-Based, Pragmatic, Randomized Controlled Trial

**DOI:** 10.2196/31401

**Published:** 2022-05-06

**Authors:** Rayner Kay Jin Tan, Wee Ling Koh, Daniel Le, Sumita Banerjee, Martin Tze-Wei Chio, Roy Kum Wah Chan, Christina Misa Wong, Bee Choo Tai, Mee Lian Wong, Alex R Cook, Mark I-Cheng Chen, Chen Seong Wong

**Affiliations:** 1 Saw Swee Hock School of Public Health National University of Singapore and National University Health System Singapore Singapore; 2 University of North Carolina Project–China Guangzhou China; 3 Action for AIDS Singapore Singapore Singapore; 4 Department of Sexually Transmitted Infections Control Clinic National Skin Centre Singapore Singapore; 5 National Skin Centre Singapore Singapore; 6 Behavioral, Epidemiological and Clinical Sciences FHI 360 Durham, NC United States; 7 National Centre for Infectious Diseases Singapore Singapore

**Keywords:** HIV, STI, testing, health promotion, eHealth, mHealth

## Abstract

**Background:**

Gay, bisexual, and other men who have sex with men (GBMSM) are at disproportionately higher risk of acquiring HIV and other sexually transmitted infections (STI). While HIV/STI testing rates among GBMSM are increasing worldwide, they remain suboptimal in a variety of settings. While many studies have attempted to evaluate the efficacy of a variety of community-based campaigns, including peer and reminder-based interventions on HIV/STI testing, however few have attempted to do so for a web drama series.

**Objective:**

This study evaluates the effectiveness of a popular web drama video series developed by a community-based organization in Singapore for GBMSM on HIV and other STI testing behaviors.

**Methods:**

The study is a pragmatic, randomized controlled trial to evaluate a popular web drama video series developed by a community-based organization in Singapore for GBMSM. A total of 300 HIV-negative, GBMSM men in Singapore aged 18 to 29 years old were recruited and block-randomized into the intervention (n=150) and control arms (n=150). Primary outcomes included changes in self-reported intention to test for, actual testing for, and regularity of testing for HIV, syphilis, chlamydia or gonorrhea, while secondary outcomes include changes in a variety of other knowledge-based and psychosocial measures at the end of the study period.

**Results:**

Overall, 83.3% (125/150) of participants in the intervention arm completed the proof of completion survey, compared to 88.7% (133/150) in the control arm. We found improvements in self-reporting as a regular (at least yearly) tester for HIV (15.9% difference, 95% CI, 3.2% to 28.6%; *P*=.02), as well as chlamydia or gonorrhea (15.5% difference, 95% CI, 4.2% to 26.9%; *P*=.009), indicating that the intervention had positively impacted these outcomes compared to the control condition. We also found improvements in participants’ intentions to test for HIV (16.6% difference, 95% CI, 4.3% to 28.9%; *P*=.009), syphilis (14.8% difference, 95% CI, 3.2% to 26.4%; *P*=.01), as well as chlamydia or gonorrhea (15.4% difference, 95% CI, 4.2% to 26.6%; *P*=.008), in the next 3 months, indicating that the intervention was effective in positively impacting intention for HIV and other STI testing among participants.

**Conclusions:**

There are clear benefits for promoting intentions to test regularly and prospectively on a broad scale through this intervention. This intervention also has potential to reach GBMSM who may not have access to conventional HIV and other STI prevention messaging, which have typically been implemented at sex-on-premises venues, bars, clubs, and in sexual health settings frequented by GBMSM. When coupled with community or population-wide structural interventions, the overall impact on testing will likely be significant.

**Trial Registration:**

ClinicalTrials.gov NCT04021953; https://clinicaltrials.gov/ct2/show/NCT04021953

**International Registered Report Identifier (IRRID):**

RR2-10.1136/bmjopen-2019-033855

## Introduction

Gay, bisexual, and other men who have sex with men (GBMSM) have been identified as key populations vulnerable to HIV acquisition [1]; however, rates of HIV testing have remained suboptimal among GBMSM in Southeast Asia. A study among young GBMSM in the Association of Southeast Asian Nations countries in 2015 found that 29.9% of participants had never had an HIV test, and these were more likely to be among younger GBMSM [2]. Unwillingness to know about their HIV status, fear of a positive result, fear of sexual orientation-related stigma or homophobia, and low perceived risk of HIV acquisition were factors found to be associated with lower rates of HIV testing among young GBMSM [3,4]. In Singapore, most individuals who test for HIV only do so through the course of medical care or through routine programmatic HIV screening, with only about 16.0% of the incident HIV cases in 2019 being diagnosed through voluntary screening. While a higher proportion (25.0%) of GBMSM tested through voluntary screening compared to heterosexual men (5.0%), diagnosis through voluntary screening remains suboptimal [5].

In general, numerous types of interventions exist that aim to increase HIV testing among GBMSM. These interventions include those that use aspects of peer education, outreach through social media, reminder-based systems, video-based interventions, and national social marketing campaigns. Social marketing campaigns have largely been promoted on a broader scale in non-Asian cities or settings where GBMSM reside [6-10], while reminder-based interventions have typically been implemented among GBMSM at sexual health clinics [11-13]. With the advent of geosocial networking smartphone apps, many interventions and campaigns now use key websites and mobile phone apps identified to be frequented by GBMSM for interventions as well [14-20].

Such interventions have reported varying degrees of effectiveness in achieving the aims of increasing HIV testing and overall disease awareness in Southeast Asia. This has been seen in the successes of the few social marketing campaigns on HIV and sexually transmitted infection (STI) testing in the region such as the “I Test, Do You?” campaign in Vietnam and the “TestXXX” campaigns in Vietnam, Thailand, the Philippines, and Indonesia [21,22].

There are, however, several limitations in the context of reach and feasibility for such interventions. For example, reminder-based and peer education-based interventions require existing health systems that can support such interventions, which may not be feasible in settings that do not have such services or where GBMSM-specific clinical services are unavailable due to the criminalization of sex between men. As such, these interventions may fall short of reaching out to more niche subsets of the GBMSM communities who may be more discreet about their sexual identities and hence may not often visit gay venues or sexual health clinics where these interventions are typically offered [23]. Furthermore, while social marketing campaigns have been effective in increasing the uptake of HIV/STI testing, such campaigns may not be feasible in settings such as Asia where negative perceptions of or attitudes toward GBMSM prevail [2].

A feasible option for interventions in such settings is the development of online, video-based interventions. However, evidence for the effectiveness of video-based interventions has not been conclusive. In a video-based intervention study conducted in Peru among GBMSM, differences in intention to test for HIV were not statistically significant between the intervention and control arm, although participants who identified as nongay did show increased willingness to do so [20]. Some studies have assessed the efficacy of crowdsourced videos on HIV testing and largely found that they were noninferior to regular health marketing campaigns [16] or had a positive effect on HIV testing rates through the use of home-based self-testing kits but not facility-based HIV/STI testing [24].

Given the gap in such research in Southeast Asia, this study sought to evaluate the effectiveness of a novel web drama series in achieving positive HIV/STI testing-related outcomes for young GBMSM. The videos used in the study form the second season of an educational and web drama miniseries, People Like Us, developed by gayhealth.sg and Action for AIDS (AFA) in 2018 [25]. The first season of the miniseries was screened at 10 film festivals and won several independent film awards. It also garnered more than 3 million views across various social media platforms since its launch in 2016. In spite of its popularity, little has been done to assess its effectiveness in positively impacting HIV and other STI testing-related outcomes. Such popular online video interventions, which have been proven to be popular and easily accessible, may complement structural interventions and allow access to underserved or hard-to-reach subgroups of GBMSM.

## Methods

### Study Aims and Design

This is a pragmatic, parallel group, randomized controlled trial (RCT) to evaluate the efficacy of a web drama series developed by a community-based organization in Singapore in increasing an individual’s intention to test, self-reported testing behaviors, and self-reported regularity of testing behaviors for HIV, syphilis, and other common STIs such as gonorrhea or chlamydia. The trial also aims to evaluate the impact of the web drama series on self-reported risk perception for HIV/STI; knowledge of risks associated with acquiring STIs and HIV; knowledge of HIV postexposure (PEP); knowledge of preexposure prophylaxis (PrEP); consistent condom use for anal sex with casual partners; incidence of STIs; connectedness to the lesbian, gay, bisexual, and transgender (LGBT) community; self-concealment of sexual orientation; perceived homophobia; internalized homophobia; HIV testing self-efficacy; and HIV testing social norms. The pragmatic nature of this trial arises due to the prospect of contamination, as the web drama series had already been launched in January 2019 and the trial was conducted among members of the community and subject to changes in the context. The implications of this are discussed later.

### Inclusion Criteria

Inclusion criteria for participants in this study include self-reporting at the point of recruitment (1) an HIV-negative status or being unsure of one’s HIV status; (2) being gay, bisexual, or queer in sexual orientation; (3) being of male gender, regardless of sex assigned at birth; (4) being aged 18 to 29 years; (5) being a Singapore citizen or permanent resident; (6) and having never watched an online video drama series by gayhealth.sg or AFA in the last year.

### Ethics Approval

The study was registered at ClinicalTrials.gov (NCT04021953). Ethical approval was provided by the National University of Singapore institutional review board (reference: S-19-059).

### Procedure and Randomization

Details of the intervention and the study procedures have been reported elsewhere in detail [26]. In brief, participants were recruited with the help of AFA and screened for eligibility through a short online survey. Throughout the entire survey process, personal identifiers were never directly linked to survey results, so as to protect the participants from potential criminal implications of disclosing their sexual activities with other men and other behaviors such as substance use. Upon completion of the enrollment survey and verification of eligibility, a staff member at AFA contacted eligible respondents to provide them with their participant ID number and formally invited them to participate in the study through the completion of the first online baseline survey. Respondents provided written consent for participation through an online participant information sheet prior to participating in the study. This survey was hosted on an encrypted, online survey administration website and took about 15 to 20 minutes to complete, and participants were reimbursed SGD 15.00 (US $10.84) for their time.

Upon completion of the baseline survey, participants were then randomly assigned in blocks of 6 in a 1:1 ratio to the intervention condition or control condition. Individuals who were assigned to the intervention condition were given a link to a series of 6 online videos, each about 10 minutes in duration, from the People Like Us web drama series, along with a link to an English-language online sexual health pamphlet tailored for GBMSM in Singapore. Individuals who were assigned to the control condition were scheduled to receive a link to the same online sexual health pamphlet as the standard of care for GBMSM at risk of acquiring HIV and other STIs in Singapore. All participants received their assigned conditions within 1 week after completing the baseline survey and were asked to complete a quiz 1 week after assignment to ascertain if participants had watched the online series of 6 videos or read the sexual health pamphlet. Participants received an SGD 20.00 (US $14.45) reimbursement following the completion of the quiz. Participants were not blinded to the group they have been assigned to and were told about their chances of being randomized to either group. At the 3-month and 6-month follow-up from the baseline, AFA contacted all eligible participants to continue with their follow-up surveys. Like the baseline survey, the second and third surveys were hosted on a survey administration website and took about 15 to 20 minutes to complete. Participants received SGD 15.00 (US $10.84) reimbursement for the completion of each survey.

### Primary Outcome Measures

The survey questionnaire can be found in Multimedia Appendix 1. Primary outcomes for this evaluation included changes in self-reported intention to test for, ever testing for, testing in the last 6 months for, and regularity of testing for HIV, syphilis, and chlamydia or gonorrhea at the 6-month postintervention follow-up. For example, participants were asked “How likely are you to get tested for HIV in the next 3 months” to which they responded using a 6-point Likert scale from extremely unlikely to get tested to extremely likely to get tested. Self-reported testing was ascertained through the question “When did you go for your last (most recent) voluntary HIV test” (options to respond include never, in the last 3 months, in the last 6 months, 6 to 12 months ago, and more than 1 year ago), while self-reported regularity of testing was measured through the question “On average, how regularly do you test for HIV” (options to respond included I do not test regularly, once every few years, once a year, once every 6 months, once every 3 months, and once a month).

### Secondary Outcome Measures

Secondary outcomes included changes in self-reported risk perception for HIV and other STIs, knowledge of HIV, knowledge of risks associated with acquiring other STIs, knowledge of HIV PEP and PrEP, self-reported consistent condom use for anal sex with casual partners (Cronbach α=.63, 95% CI, 0.62 to 0.65), self-reported incidence of STIs, and other scales validated among GBMSM in other settings. These included scale measurements of connectedness to the LGBT community (Cronbach α=.87, 95% CI, 0.86 to 0.87) [27], self-concealment of sexual orientation (Cronbach α=.90, 95% CI, 0.90 to 0.91) [28], outness inventory (Cronbach α=.77, 95% CI, 0.77 to 0.79) [29], relevance of sexual orientation disclosure to sexual health care providers (Cronbach α=.83, 95% CI, 0.82 to 0.83), perceived homophobia (Cronbach α=.84, 95% CI, 0.83 to 0.84), internalized homophobia (Cronbach α=.85, 95% CI, 0.85 to 0.85) [30], HIV testing self-efficacy (Cronbach α=.90, 95% CI, 0.90 to 0.91) [31], and HIV testing social norms (Cronbach α=.55, 95% CI, 0.53 to 0.57) [32].

### Sample Size

As the primary outcome of interest included HIV or other STI testing in the last 3 months, we used data from a previous study among 1098 GBMSM recruited through Grindr, the popular geosocial networking app [23,33]. The study found that 50.4% of respondents reported having had an HIV test in the 6 months prior to the survey. Assuming a 50% increase in recent HIV testing as a result of the intervention, as data from previous studies based on the impact such a web drama series on recent HIV testing remains limited [34], a sample size of 112 in each arm will yield statistical power higher than 80% to detect a significant change for the intervention based on calculations generated by a web-based sample size calculator (ClinCalc LLC) software. A target sample size of 150 participants per group was proposed to account for an attrition rate of 25% for each group across the 6-month follow-up.

### Statistical Analyses

The baseline sociodemographic characteristics and primary outcome variables in the intervention and control groups were compared and any between-group differences were determined through chi-square tests. Intervention efficacy was analyzed over the entire study period (from baseline to the 6-month assessment) via chi-square tests for primary outcomes and Wilcoxon rank-sum tests for secondary outcomes with continuous variables, with median and interquartile ranges (IQR) also reported. Nonparametric tests were used for our analyses [35]. All analyses were evaluated based on the principle of intention to treat. While a 2-sided test at the 5% level of significance was indicated in our original protocol, we will discuss degrees of evidence in our paper instead [36]. We used the statistical software Stata (version 15, StataCorp LLC).

## Results

### Participant Characteristics

**Figure 1:**
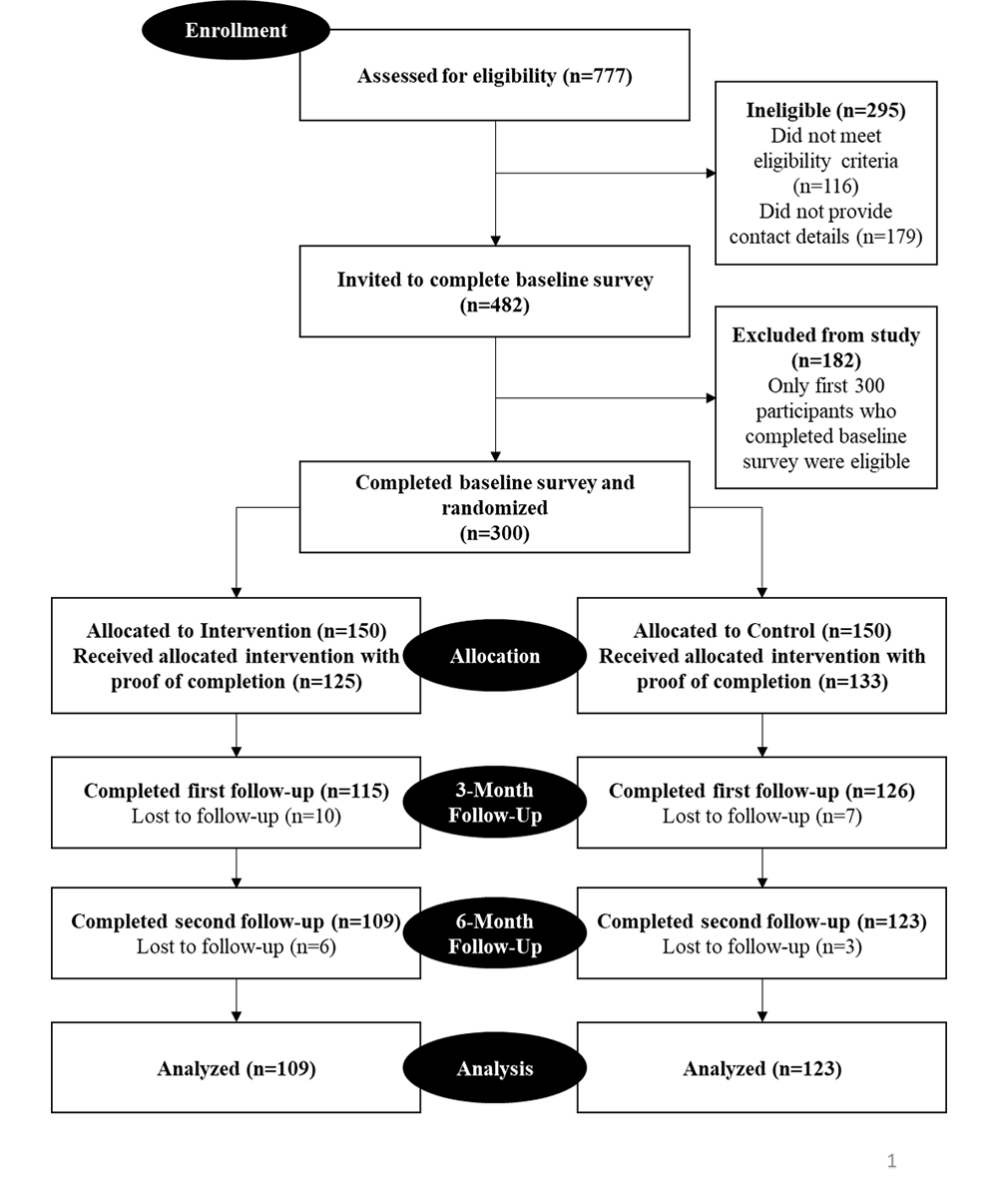
Consolidated Standards of Reporting Trials diagram.

Overall, 777 participants were assessed for eligibility, and 482 participants were invited to participate to complete the baseline survey; 179 participants did not provide any contact details for follow-up, and 116 of them did not meet the eligibility criteria of being of male gender; identifying as gay, bisexual, or queer; and self-reporting as being HIV-negative. Overall, 83.3% (125/150) of participants in the intervention arm completed the proof of completion survey compared to 88.7% (133/150) in the control arm. At the first follow-up at 3 months, 8.0% (10/125) of participants in the intervention arm and 5.3% (7/133) in the control arm were lost to follow-up; at the second follow-up at 6 months, 5.2% (6/115) of participants in the intervention arm and 2.4% (3/126) in the control arm were lost to follow-up. Overall cumulative attrition rates reported for the intervention and control arms were 27.3% and 18.0%, respectively. The CONSORT (Consolidated Standards of Reporting Trials) diagram for the study is shown in Figure 1.

Table 1 summarizes patient demographic characteristics and outcome measures at baseline for the control and intervention groups. Overall, 150 participants were randomized to each arm. The 2 groups were generally well-balanced across all sociodemographic and baseline outcomes measures except for a slight imbalance for type of housing, where more participants in the intervention arm (126/150, 84.0%) reported staying in public housing relative to those in the control arm (112/150, 74.7%).

**Table 1:** Demographic characteristics and outcome variables for control and intervention groups.

Demographic and outcome variables at baseline			Control (n=150)		Intervention (n=150)
**Demographic variables**					
	Age (year), mean (SD)	23.8 (2.99)		24.0 (2.98)	
	Chinese (ref: non-Chinese), n (%)	124 (82.7)		113 (75.3)	
	Gay (ref: bisexual, queer, or other), n (%)	127 (84.7)		116 (77.3)	
	Educational attainment below college (ref: some college), n (%)	95 (63.3)		92 (61.3)	
	Public housing (ref: private housing), n (%)	112 (74.7)		126 (84.0)	
	Monthly income below SGD^a^ 5000 (ref: SGD 5000 and above), n (%)	82 (54.7)		73 (48.7)	
**Outcome variables**					
	Ever tested for HIV, n (%)	113 (75.3)		108 (72.0)	
	Ever tested for syphilis, n (%)	73 (48.7)		77 (51.3)	
	Ever tested for chlamydia or gonorrhea, n (%)	55 (36.7)		59 (39.3)	
	Tested for HIV in last 6 months, n (%)	66 (44.0)		63 (42.0)	
	Tested for syphilis in last 6 months, n (%)	39 (26.0)		41 (27.3)	
	Tested for chlamydia or gonorrhea in last 6 months, n (%)	21 (14.0)		26 (17.3)	
	Tested regularly (at least yearly) for HIV, n (%)	68 (45.3)		68 (45.3)	
	Tested regularly (at least yearly) for syphilis, n (%)	37 (24.7)		44 (29.3)	
	Tested regularly (at least yearly) for chlamydia or gonorrhea, n (%)	28 (18.7)		34 (22.7)	
	Intention to test for HIV in the next 3 months, n (%)	56 (37.3)		59 (39.3)	
	Intention to test for syphilis in the next 3 months, n (%)	41 (27.3)		39 (26.0)	
	Intention to test for chlamydia or gonorrhea in the next 3 months, n (%)	33 (22.0)		33 (22.0)	

^a^SGD: Singapore dollar.

### Primary Outcomes: HIV and Other STI Testing

**Figure 2:**
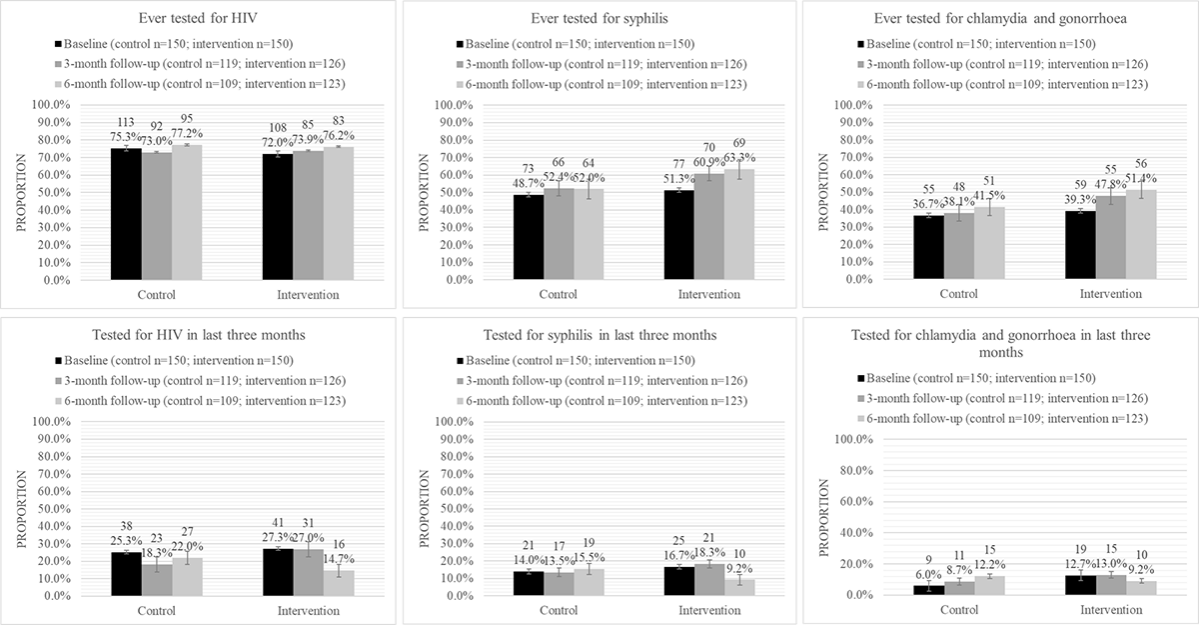
Trends in ever testing and recent testing for HIV and other sexually transmitted infections in control and intervention arms. High-resolution version of the figure is in Multimedia Appendix 2.

**Figure 3:**
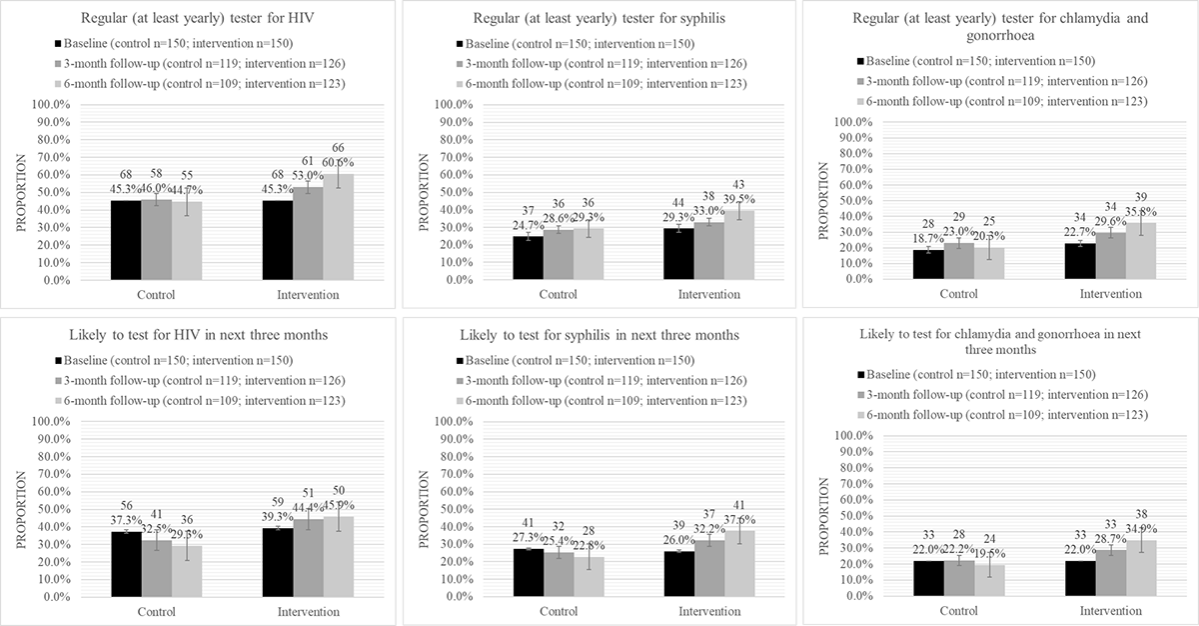
Trends in being a regular (at least yearly) tester and intention to test for HIV and other sexually transmitted infections in control and intervention arms. High-resolution version of the figure is in Multimedia Appendix 3.

We compared the primary outcomes for the intervention and control groups at 6 months postintervention, specifically, for ever testing, recent testing in the past 6 months, testing regularly (at least yearly), and intention to test in the next 3 months for HIV, syphilis, and chlamydia or gonorrhea. These findings are summarized in Table 2 and Figures 2 and 3. We observed marginal increases in recent testing for HIV, syphilis, and chlamydia or gonorrhea in the last 6 months. We also observed larger increases in the rates of ever testing for syphilis (11.3% difference, 95% CI, –1.4% to 24.0%) and for chlamydia and gonorrhea (9.9%, 95% CI, –2.9% to 22.7%).

We found the greatest improvements in self-reporting for testing regularly (at least yearly) for HIV (15.9% difference, 95% CI, –28.6% to –3.2%) and for chlamydia or gonorrhea (15.5% difference, 95% CI, –26.9% to –4.2%), indicating that the intervention had positively impacted these outcomes compared to the control condition. We also found greatest improvements in participants’ intentions to test for HIV (16.6% difference, 95% CI, –28.9% to –4.3%), syphilis (14.8% difference, 95% CI, –26.4% to –3.2%), and chlamydia or gonorrhea (15.4% difference, 95% CI, –26.6% to –4.2%) in the next 3 months, indicating that the intervention had positively impacted intention for HIV and other STI testing among participants in the intervention arm vis-à-vis those in the control arm.

**Table 2:** Comparison of primary outcome measures at 6 months postintervention.

Primary outcome variables	Control (n=123), n (%)	Intervention (n=109), n (%)	Difference, %	95% CI	*P* value
Ever tested for HIV	95 (77.2)	83 (76.2)	–1.1	–11.9 to 9.9	.85
Ever tested for syphilis	64 (52.0)	69 (63.3)	11.3	–1.4 to 24.0	.08
Ever tested for chlamydia and gonorrhea	51 (41.5)	56 (51.4)	9.9	–2.9 to 22.7	.13
Tested for HIV in last 6 months	41 (33.3)	42 (38.5)	5.2	–7.1 to 17.5	.41
Tested for syphilis in last 6 months	31 (25.2)	30 (27.5)	2.3	–9.0 to 13.6	.69
Tested for chlamydia and gonorrhea in last 6 months	22 (17.9)	25 (22.9)	5.1	–5.3 to 15.3	.34
Tested regularly (at least yearly) for HIV	55 (44.7)	66 (60.6)	15.8	3.2 to 28.6	.02
Tested regularly (at least yearly) for syphilis	36 (29.3)	43 (39.5)	10.2	–2.0 to 22.4	.10
Tested regularly (at least yearly) for chlamydia and gonorrhea	25 (20.3)	39 (35.8)	15.5	4.2 to 26.9	.009
Intention to test for HIV in the next 3 months	36 (29.3)	50 (45.9)	16.6	4.3 to 28.9	.009
Intention to test for syphilis in the next 3 months	28 (22.8)	41 (37.6)	14.8	3.2 to 26.4	.01
Intention to test for chlamydia and gonorrhea in the next 3 months	24 (19.5)	38 (34.9)	15.4	4.2 to 26.6	.008

### Secondary Outcomes: HIV and Other STI Testing

We compared the secondary outcomes for the intervention and control groups at 6 months postintervention; specifically, perceived risk for HIV and other STIs, knowledge of HIV and other STIs, knowledge of HIV PEP and PrEP, inconsistent condom use for anal sex with casual partners in the last 6 months, recent STI diagnoses in the last 6 months, connectedness to the LGBT community, sexual orientation concealment, outness inventory, perceived relevance of sexual orientation disclosure in sexual health care, perceived homophobia, internalized homophobia, HIV testing self-efficacy, and HIV testing social norms. These findings are summarized in Table 3. Marginal differences in all secondary outcomes were observed.

**Table 3:** Comparison of secondary outcome measures at 6 months postintervention.

Secondary outcome variables	Control (n=123), median (IQR)	Intervention (n=109), median (IQR)	Difference^a^, %	95% CI^a^	*P* value^b^
Perceived HIV risk, median (IQR)	2.0 (15.0)	6.0 (20.0)	0	0-0	.32
Perceived risk of other sexually transmitted infections, median (IQR)	5.0 (20.0)	8.0 (25.0)	0	0-0	.49
HIV and other sexually transmitted infections knowledge, median (IQR)	6.0 (2.0)	6.0 (1.0)	0	0-0	.08
Knowledge of HIV postexposure prophylaxis, n (%)	113 (91.9)	103 (94.5)	2.6	–3.9 to 9.1	.43
Knowledge of HIV preexposure prophylaxis, n (%)	122 (99.2)	107 (98.2)	–1.0	–3.9 to 1.9	.49
Inconsistent condom use in last 6 months with casual partners, n (%)	20 (16.3)	15 (13.8)	–2.5	–11.7 to 6.7	.60
Incidence of sexually transmitted infections in last 6 months, n (%)	8.0 (6.5)	4.0 (3.7)	–2.8	–8.4 to 2.8	.33
Connectedness to the lesbian, gay, bisexual, and transgender community, median (IQR)	22.0 (5.0)	21.0 (6.0)	1	–2.0 to 1.0	.35
Self-concealment of sexual orientation, median (IQR)	19.0 (10.0)	20.0 (9.0)	–1.0	–3.0 to 1.0	.30
Outness inventory, median (IQR)	2.74 (1.88)	2.33 (1.88)	0.1	–0.2 to 0.4	.45
Disclosure of sexual orientation to health care provider, median (IQR)	25.0 (8.0)	23.0 (9.0)	1	0 to 3.0	.17
Perceived homophobia, median (IQR)	18.0 (4.0)	18.0 (4.0)	0	–1.0 to 1.0	.94
Internalized homophobia, median (IQR)	10.0 (6.0)	10.0 (5.0)	0	–1.0 to 1.0	.56
HIV self-testing efficacy, median (IQR)	38.0 (11.0)	36.0 (11.0)	0	–2.0 to 2.0	.76
HIV testing social norms, median (IQR)	22.0 (4.0)	22.0 (4.0)	0	–1.0 to 0	.28

^a^Median differences between the groups and 95% CIs were estimated with the Hodges-Lehmann method.

^b^*P* value is derived from the Wilcoxon rank-sum test.

## Discussion

### Principal Findings

This study was a pragmatic, community-based, RCT on the effectiveness of the second season of the People Like Us web drama series. We found that the web drama series coupled with sexual health information provided through a pamphlet was more effective in promoting participants’ self-reported intentions to test regularly (at least yearly) for chlamydia or gonorrhea and intentions to test for HIV and other STIs compared to the control condition that involved the availability of the pamphlet only. The intervention was developed to increase viewers’ knowledge and perceptions of HIV and other STI risk, address homophobia and sexual orientation disclosure, increase safer-sex negotiation self-efficacy, promote positive attitudes toward condom use and other safe sex behaviors, build skills and self-efficacy for practicing safer sex, provide information on HIV and other STI testing and its benefits, provide information on resources for HIV and other STI testing and other mental health services, and model appropriate behaviors around practicing safer sex. As such, we hypothesized that the intervention would be more effective than traditional sexual health pamphlets in positively impacting a range of primary outcomes around HIV and other STI testing and secondary outcomes around HIV and other STI risk, safer sex, homophobia, HIV testing self-efficacy, and social norms.

The intervention was effective in promoting intentions to test for HIV and other STIs among participants in the intervention group compared to controls and, to a lesser degree, promoting ever testing for other STIs throughout the study period. We observed that recent testing for HIV and other STIs had also increased at the 3-month follow-up. We hypothesize that the lack of a strong impact of the intervention on actual testing variables may be attributed to the following reasons. The first reason, we hypothesize, may be attributed to the COVID-19 pandemic; the implementation of movement controls and legislation affecting social and sexual behaviors have caused an overall decrease in the rates of testing during the trial period. Given the descriptive increase in ever testing for other STIs and increase in recent testing for HIV and other STIs at the 3-month follow-up, there is reason to believe that this might have led to the marginal impact of the intervention for these variables. Second, while the intervention sought to potentially address individual beliefs around testing, it was not as effective in addressing complex psychosocial constructs such as perceived or internalized homophobia, self-efficacy, and social norms around testing, which were measured as secondary outcomes of the study and also serve as important constructs that underpin eventual testing behaviors [37-40]. These complex and often deep-rooted psychological constructs have been successfully modified in other types of more complex interventions that typically comprise components of training and engagement, individual counselling, and peer engagement [41-43]. Finally, the lack of impact of the intervention on actual testing variables may be due to the impact of contamination in the study. At the end of the study, 13.8% (17/123) of participants in the control group had seen the People Like Us series and, in other words, had been exposed to the intervention prior to this study. As such, this may have biased the effect of the intervention toward the null. Unfortunately, the same question was not asked among participants who were in the intervention group, and we cannot ascertain if the dilution of the intervention effect might have occurred due to perhaps a high rate of exposure to the intervention prior to the study as well.

### Pragmatic Nature of Trial

The People Like Us web drama series was launched in the community prior to the start of this study, and thus members of the community might have been exposed to the intervention prior to the study. Furthermore, researchers did not have an opportunity to participate in the development of the proposed intervention, and thus the intervention was not developed with a predetermined theory of behavior change. However, this study was designated to continue in view of its importance in the local context to evaluate the efficacy of such web drama series and justify further HIV and other STI prevention efforts that use online channels.

Given the pragmatic nature of the trial as described above, there was a possibility that control group participants may be exposed to the video series during the 6-month study period. To mitigate this, we ensured that details of the online video intervention (ie, title of web series, where to access it) were not included in the participant information sheet—only basic information on the possibility that they may be randomized to an online video intervention was mentioned. Furthermore, to reduce the possibility of contamination occurring in reaction to being asked the screening question, we avoided using the title of the web series but instead asked the question “Have you watched an online video drama series filmed by gayhealth.sg or AFA Singapore in the past year,” as this is gayhealth.sg and AFA’s only web series launched in the past year. While the generic nature of the question may have resulted in underreporting of viewing the video series, all participants eventually reported if they had viewed any of the episodes prior to or during the study period.

Specifically, participants in the treatment group were asked if they had previously watched any of the episodes when they submitted the intervention completion survey 1 week after the completion of their baseline survey, while the control group received a link to all 6 episodes of the video intervention alongside their final survey at the 6-month mark and were asked specifically which episodes they had watched prior to or during the intervention period.

The study team relied on self-reported outcomes such as testing behaviors and HIV/STI diagnoses as it is presently not possible to link clinic attendance or laboratory-confirmed diagnostic tests for HIV and other STIs to individual participants. These issues have arisen due to ethical concerns around linking participants’ personal information to survey results, which collect information on criminalized behavior such as sexual intercourse with other men, among participants in the sample. However, the findings of this proposed study would serve as a proof-of-concept for future studies that may be able to obtain funding and state support for other means of testing, such as the use of self-testing kits for HIV and other STIs.

### Strengths and Limitations

The People Like Us web drama series has had vast reach among GBMSM in Singapore and around the world. As of April 2022, it has been nominated for the 48th International Emmy Awards for Best Short-Form Series and acquired by cable television and over-the-top media platforms such as HereTV, Gagaoolala, and Dekko and has attained more than 3 million views on YouTube. Given the viral nature of this web drama series, coupled with its vast reach among GBMSM, we believe that there are clear benefits to the promotion of such interventions in the GBMSM community. While it may not be able to address more complex constructs that underpin testing, there are clear benefits for promoting intentions to test regularly and prospectively for a wide audience; when coupled with community- or population-wide structural interventions, the overall impact on testing will be significant. Second, the nature of the web drama series allows this intervention to reach GBMSM who may not have access to conventional HIV and other STI prevention messaging that have typically been implemented at sex-on-premises venues, bars, clubs, and in sexual health settings frequented by GBMSM.

### Limitations

We are also mindful of several limitations in this trial. First, given the pragmatic nature of the trial, we could not control for external situations such as the COVID-19 pandemic. As such, several aspects of the trial could not be evaluated in a manner that was intended and effects of the intervention may be underestimated in several respects. Second, contamination was a potential issue, where we found that at the end of the trial, 13.8% (17/123) of participants who remained in the control arm had seen the People Like Us season 2 series in spite of the initial screening question. However, intention-to-treat analysis was conducted, thus reducing the risk of overstating the effectiveness of the trial. Furthermore, given that most video-based interventions have been shown to largely influence short-term health behavior change rather than sustained, long-term behaviors [44], the potential decay and wash-out period for the intervention among these participants is less likely to have a substantial impact on the study results. Third, given the resource limitations of this study, we were not able to ascertain actual behaviors for HIV and other STI testing among participants through clinic attendance, and such measures were instead self-reported; however, we believe that the impact of any recall bias may have been minimal due to the introduction of the 3-month follow-up period between the pre- (baseline) and postintervention time points (6-month follow-up). Last, a formal process evaluation of the trial was not conducted due to a lack of resources, and thus we were not able to generate deeper insight into issues of implementation fidelity of the trial. Future trials should incorporate qualitative approaches to enhance our understanding of the individual and structural mechanisms that have led to effectiveness of the intervention among participants.

Nevertheless, this trial was conducted through a pilot study grant and will help to better inform future process evaluation efforts on larger trial studies similar in design. The pragmatic nature of the trial also meant that the trial was conducted in a community-based setting and subject to broader changes in the context in which it was rolled out. However, it was also during this time that COVID-19 started to take its hold as a pandemic, with the first case reported in Singapore on January 23, 2020. Further details on how this may have impacted the trial can be found in Multimedia Appendix 4 (see Figure S1 and Table S1 with explanatory notes).

### Recommendations and Conclusions

Overall, this pragmatic, community-based RCT found that the critically acclaimed, wide-reaching People Like Us web drama series was effective in driving intentions to test regularly and prospectively for HIV and other STIs. We have several recommendations that accompany these findings. First, we recommend scaling up this intervention to a wider audience through further support and funding for marketing and promotional efforts, as this will help drive greater intentions to test among GBMSM, including those who may not be reached through traditional channels of sexual health communications and marketing. Second, we recommend rolling out additional programs alongside the marketing of the web drama series that address structural barriers to testing, such as issues of access and cost of testing, in the form of testing coupons or vouchers. Information on free and anonymous testing in the local GBMSM community by nongovernmental organizations such as AFA Singapore should be made available. Additionally, programs and workshops addressing deeply rooted, complex psychological constructs should also be offered to participants alongside the marketing of the web drama series to further drive HIV and other STI testing and related HIV prevention health behaviors. These may include workshops specifically on risks associated with HIV and other STIs, negotiating sexual relationships, and addressing homophobia and other topics underlying individual beliefs around HIV and other STIs that may underpin HIV and other STI testing behaviors.

Future trials and interventions should focus on addressing the limitations of this study. First, free clinic-based testing should be provided as outcomes for the trial to simultaneously address issues of structural barriers and limitations in outcome measurement. Second, the intervention should be structured to provide more complex, internet-based components such as online workshops, counselling, or peer support structures that may directly address the more complex, secondary outcomes of the study. Third, subgroup analyses to explore the impact of different demographic factors on intervention effectiveness can be conducted to further nuance and inform differentiated service delivery models targeting the rollout of such interventions for GBMSM. Finally, future studies should consider the potential impact that COVID-19 health promotion messaging, such as vaccinations and prevention methods, may have on HIV and other STI testing promotion in the community.

## Appendix

Multimedia Appendix 1 Survey questionnaire.

Multimedia Appendix 2 High-resolution version of Figure 2.

Multimedia Appendix 3 High-resolution version of Figure 3.

Multimedia Appendix 4 COVID-19 and its impact on the study.

Multimedia Appendix 5 CONSORT-eHEALTH checklist (V 1.6.1).

## Abbreviations

AFA: Action for AIDS
CONSORT: Consolidated Standards of Reporting Trials
GBMSM: gay, bisexual and other men who have sex with men
LGBT: lesbian, gay, bisexual, and transgender
PEP: HIV postexposure prophylaxis
PrEP: HIV preexposure prophylaxis
RCT: randomized controlled trial
SGD: Singapore dollar
STI: sexually transmitted infection
