# Supplementary material for: Effect of a Popular Web Drama Video Series on HIV and Other Sexually Transmitted Infection Testing Among Gay, Bisexual, and Other Men Who Have Sex With Men in Singapore: Community-Based, Pragmatic, Randomized Controlled Trial
Source: J Med Internet Res. 2022 May 6;24(5):e31401. doi: 10.2196/31401 (PMC9123545; doi:10.2196/31401)
Supplement: Multimedia Appendix 1 [file jmir_v24i5e31401_app1.docx]

**People Like Us**

**Baseline sociodemographic measures**

| **#** | **Question** | **Response categories** | |
| --- | --- | --- | --- |
|  | How old are you? | [Open-ended response] | |
|  | What sexual orientation do you identify yourself with the most? | Straight / Heterosexual  Gay / Homosexual  Bisexual  Others: ___________________ | |
|  | What is your residence status? | **Please select all that apply:**  Singaporean citizen  Singapore permanent resident  Others (please specify): ________________________ | |
|  | How do you identify yourself ethnically? | Chinese  Malay  Indian  Others (please specify): *________________________* | |
|  | What is your religion? | Buddhism  Islam  Hinduism  Christianity  Taoism | Sikhism  Agnostic  Atheist  Others (Please specify):  _____________________ |
|  | What is the highest level of education you have completed? | Primary Education  Secondary Education  GCE ‘N’ Levels or equivalent  GCE ‘O’ Levels or equivalent  GCE ‘A’ Levels or equivalent  Professional Certificate  Diploma  Bachelor’s Degree  Postgraduate Degree  Others _(10)_ (please specify): *________________________* | |
|  | Are you currently working? | Employed, please specify job:  Unemployed  Retired  Student  Other, please specify: | |
|  | What type of housing are you staying in? | 1 Room HDB  2 Room HDB  3 Room HDB  4 Room HDB  5 Room HDB  Maisonette or other executive public housing  Condominium  Terrace, Bungalow, and other Private Landed Property  Others: ___________________ | |
|  | On average, what is your gross personal monthly income (before CPF and tax deductions, if any)? | Not earning an income  < SGD1000  SGD1000 – SGD1999  SGD2000 – SGD2999  SGD3000 – SGD3999  SGD4000 – SGD4999  SGD5000 and above | |
|  | What is your current relationship status? | Single  Partnered, with single male partner  Partnered, with multiple male partners  Partnered, with single female partner  Partnered, with multiple female partners  Partnered, with multiple male and female partners | |
|  | At what age did you first have a sexual experience involving oral sex? | [Open-ended response] | |
|  | At what age did you first have a sexual experience involving anal sex? | [Open-ended response] | |

**Primary outcome measures**

| **HIV/STI Testing Behaviors** | | |
| --- | --- | --- |
|  | When did you go for you last (most recent) voluntary HIV test? | Never  In the last 3 months  In the last 6 months  6 to 12 months ago  More than 1 year ago |
|  | When did you go for you last (most recent) voluntary Syphilis test? | Never  In the last 3 months  In the last 6 months  6 to 12 months ago  More than 1 year ago |
|  | When did you go for you last (most recent) voluntary Chlamydia or Gonorrhea test? | Never  In the last 3 months  In the last 6 months  6 to 12 months ago  More than 1 year ago |
|  | How much risk do you think you are at of getting HIV? | Slider scale: 0 to 10 (0=no risk at all; 10=very high risk) |
|  | How much risk do you think you are at of getting other sexually transmitted infections? | Slider scale: 0 to 10 (0=no risk at all; 10=very high risk) |
|  | How likely are you to get tested for HIV in the next three months? | Extremely unlikely to get tested  Very unlikely to get tested  Somewhat unlikely to get tested  Somewhat likely to get tested  Very likely to get tested  Extremely likely to get tested |
|  | How likely are you to get tested for Syphilis in the next three months? | Extremely unlikely to get tested  Very unlikely to get tested  Somewhat unlikely to get tested  Somewhat likely to get tested  Very likely to get tested  Extremely likely to get tested |
|  | How likely are you to get tested for Chlamydia or Gonorrhea in the next three months? | Extremely unlikely to get tested  Very unlikely to get tested  Somewhat unlikely to get tested  Somewhat likely to get tested  Very likely to get tested  Extremely likely to get tested |
|  | On average, how regularly do you test for HIV? | I do not test regularly  Once every few years  Once a year  Once every 6 months  Once every 3 months  Once a month  Others (please specify): ______________________ |
|  | On average, how regularly do you test for Syphilis? | I do not test regularly  Once every few years  Once a year  Once every 6 months  Once every 3 months  Once a month  Others (please specify): ______________________ |
|  | On average, how regularly did you test for other STDs or STIs that are not HIV or Syphilis? | I do not test regularly  Once every few years  Once a year  Once every 6 months  Once every 3 months  Once a month  Others (please specify): ______________________ |
|  | Where do you typically get your voluntary HIV test done? You may choose more than one answer. | Government-Run Clinic (e.g. DSC Clinic)  Government-Run Hospital (e.g. General hospital)  GP Clinic (Regular testing hours)  GP Clinic (Anonymous testing hours)  Anonymous Test Site (NGO: Action for AIDS)  Overseas (please specify where): ______________  Others (please specify): ______________________ |
|  | Where do you typically get your voluntary Syphilis test done? You may choose more than one answer. | Government-Run Clinic (e.g. DSC Clinic)  Government-Run Hospital (e.g. General hospital)  GP Clinic (Regular testing hours)  GP Clinic (Anonymous testing hours)  Anonymous Test Site (NGO: Action for AIDS)  Overseas (please specify where): ______________  Others (please specify): ______________________ |
|  | Where do you typically get your voluntary test for STDs or STIs that are not HIV or Syphilis? You may choose more than one answer. | Government-Run Clinic (e.g. DSC Clinic)  Government-Run Hospital (e.g. General hospital)  GP Clinic (Regular testing hours)  GP Clinic (Anonymous testing hours)  Anonymous Test Site (NGO: Action for AIDS)  Overseas (please specify where): ______________  Others (please specify): ______________________ |

**Secondary Outcome Measures**

| **Knowledge about HIV and STI Prevention** | | | | | | | | | | | | | | | | | | | |
| --- | --- | --- | --- | --- | --- | --- | --- | --- | --- | --- | --- | --- | --- | --- | --- | --- | --- | --- | --- |
|  | HIV Pre-Exposure Prophylaxis is an effective means of preventing HIV infection | True  False  I do not know | | | | | | | | | | | | | | | | | |
|  | HIV Pre-Exposure Prophylaxis can also reduce the chances of acquiring other STIs | True  False  I do not know | | | | | | | | | | | | | | | | | |
|  | Gonorrhea can be transmitted through oral sex | True  False  I do not know | | | | | | | | | | | | | | | | | |
|  | There is a/are clinics in Singapore where I can test anonymously for HIV and Syphilis | True  False  I do not know | | | | | | | | | | | | | | | | | |
|  | Chemsex, or the use of substances during sex, increases a person’s risk of engaging in risky sexual behavior and acquiring HIV or other STIs | True  False  I do not know | | | | | | | | | | | | | | | | | |
|  | An HIV-positive individual who has achieved viral suppression or an ‘undetectable’ viral load has almost zero chances of transmitting the virus to someone else through sexual intercourse | True  False  I do not know | | | | | | | | | | | | | | | | | |
|  | An HIV-positive individual on effective treatment can live a long, healthy, and productive life | True  False  I do not know | | | | | | | | | | | | | | | | | |
| **Connectedness to LGBT Community Scale** | | | | | | | | | | | | | | | | | | | |
|  | **These are questions about the LGBT community in Singapore. By LGBT community, we don't mean any particular group, but in general the wider community of LGBTQ people.** | **Strongly Disagree** | | | | **Disagree** | | | | | **Agree** | | | | **Strongly Agree** | | | | |
|  | You feel you are a part of Singapore’s LGBT community |  | | | |  | | | | |  | | | |  | | | | |
|  | Participating in Singapore’s LGBT community is a positive thing for you |  | | | |  | | | | |  | | | |  | | | | |
|  | You feel a bond with the LGBT community |  | | | |  | | | | |  | | | |  | | | | |
|  | You are proud of Singapore’s LGBT community |  | | | |  | | | | |  | | | |  | | | | |
|  | It is important for you to be politically active in Singapore’s LGBT community |  | | | |  | | | | |  | | | |  | | | | |
|  | If we work together, LGBT people can solve problems in Singapore’s LGBT community |  | | | |  | | | | |  | | | |  | | | | |
|  | You really feel that any problems faced by Singapore’s LGBT community are also your own problems |  | | | |  | | | | |  | | | |  | | | | |
|  | You feel a bond with other gay, bisexual, or queer men |  | | | |  | | | | |  | | | |  | | | | |
| **Outness Inventory** | | | | | | | | | | | | | | | | | | | |
| **Use the following rating scale to indicate how open you are about your sexual orientation to the people listed below. Try to respond to all of the items, but leave items blank if they do not apply to you.**  1 = person definitely does NOT know about your sexual orientation status  2 = person might know about your sexual orientation status, but it is NEVER talked about  3 = person probably knows about your sexual orientation status, but it is NEVER talked about  4 = person probably knows about your sexual orientation status, but it is RARELY talked about  5 = person definitely knows about your sexual orientation status, but it is RARELY talked about  6 = person definitely knows about your sexual orientation status, and it is SOMETIMES talked about  7 = person definitely knows about your sexual orientation status, and it is OPENLY talked about  0 = not applicable to your situation; there is no such person or group of people in your life | | | | | | | | | | | | | | | | | | | |
|  | Mother | 1 | 2 | | | 3 | 4 | | | | 5 | | | 6 | | 7 | | 0 | |
|  | Father | 1 | 2 | | | 3 | 4 | | | | 5 | | | 6 | | 7 | | 0 | |
|  | Siblings (Sisters, Brothers) | 1 | 2 | | | 3 | 4 | | | | 5 | | | 6 | | 7 | | 0 | |
|  | Extended family / relatives | 1 | 2 | | | 3 | 4 | | | | 5 | | | 6 | | 7 | | 0 | |
|  | My new straight friends | 1 | 2 | | | 3 | 4 | | | | 5 | | | 6 | | 7 | | 0 | |
|  | My work peers | 1 | 2 | | | 3 | 4 | | | | 5 | | | 6 | | 7 | | 0 | |
|  | My work supervisor(s) | 1 | 2 | | | 3 | 4 | | | | 5 | | | 6 | | 7 | | 0 | |
|  | Members of my religious community (e.g. church, temple) | 1 | 2 | | | 3 | 4 | | | | 5 | | | 6 | | 7 | | 0 | |
|  | Leaders of my religious community (e.g. church, temple) | 1 | 2 | | | 3 | 4 | | | | 5 | | | 6 | | 7 | | 0 | |
|  | Strangers, new acquaintances | 1 | 2 | | | 3 | 4 | | | | 5 | | | 6 | | 7 | | 0 | |
|  | My old (long-time) heterosexual friends | 1 | 2 | | | 3 | 4 | | | | 5 | | | 6 | | 7 | | 0 | |
|  | General healthcare professionals (e.g. general practitioners, family doctors) | 1 | 2 | | | 3 | 4 | | | | 5 | | | 6 | | 7 | | 0 | |
|  | Sexual healthcare professionals (e.g. specialist sexual health doctors and nurses) | 1 | 2 | | | 3 | 4 | | | | 5 | | | 6 | | 7 | | 0 | |
| **Modified Self-Concealment Scale** | | | | | | | | | | | | | | | | | | | |
|  | **Below are a list of statements regarding your perceptions towards sex. Please indicate the answer that best describes your answer to each statement.** | **Strongly Disagree** | | | **Disagree** | | | | **Neither Agree nor Disagree** | | | | **Agree** | | | | **Strongly Agree** | | |
|  | I haven’t shared with anyone that I  have sex with men |  | | |  | | | |  | | | |  | | | |  | | |
|  | If I shared with my friends that I have  sex with men, they would like me less |  | | |  | | | |  | | | |  | | | |  | | |
|  | There are lots of things about my sex  with men that I keep to myself |  | | |  | | | |  | | | |  | | | |  | | |
|  | When I have sex with men, I keep it to  myself |  | | |  | | | |  | | | |  | | | |  | | |
|  | I would lie if anyone asked me if I  have sex with men |  | | |  | | | |  | | | |  | | | |  | | |
|  | The fact that I have sex with men is  too embarrassing to share with others |  | | |  | | | |  | | | |  | | | |  | | |
|  | I have thoughts about my sex with  men that I never share with anyone |  | | |  | | | |  | | | |  | | | |  | | |
| **Sexual Orientation Disclosure in Healthcare** | | | | | | | | | | | | | | | | | | | |
|  | **Below are a list of statements regarding your perceptions towards sexual healthcare providers. Please indicate the answer that best describes your answer to each statement.** | **Strongly Disagree** | | | **Disagree** | | | | **Neither Agree nor Disagree** | | | | **Agree** | | | | **Strongly Agree** | | |
|  | My sexual orientation is relevant to my sexual health |  | | |  | | | |  | | | |  | | | |  | | |
|  | It is important that my sexual healthcare provider knows about my sexual orientation |  | | |  | | | |  | | | |  | | | |  | | |
|  | I feel comfortable letting my sexual healthcare provider know about my sexual orientation |  | | |  | | | |  | | | |  | | | |  | | |
|  | I am likely to disclose my sexual orientation to my sexual healthcare provider during my next visit to the clinic |  | | |  | | | |  | | | |  | | | |  | | |
|  | I feel that I would be stigmatised or discriminated against if I were to disclose my sexual orientation to my healthcare provider |  | | |  | | | |  | | | |  | | | |  | | |
|  | I feel that others might find out about my sexual orientation if I were to tell my sexual healthcare provider about my sexual orientation |  | | |  | | | |  | | | |  | | | |  | | |
|  | I worry that my sexual orientation will be shared with other organisations if I were to disclose my sexual orientation to my sexual healthcare provider |  | | |  | | | |  | | | |  | | | |  | | |
| **Sexual Risk Behaviors** | | | | | | | | | | | | | | | | | | | |
|  | In the last 3 months, how many different male partners did you have sex with (oral OR anal sex)? | 0  1  2  3  4  5-9 | | | | | | | | 10-14  15-19  20-24  25-29  30 or more | | | | | | | | | |
|  | In the last 3 months, were you diagnosed with, or treated for any of the following sexually transmitted infections? | **Please select all that apply:**  HIV  Gonorrhea  Syphilis  Chlamydia  Genital Herpes  Genital Warts  Hepatitis C  Others (please specify): ______________________ | | | | | | | | | | | | | | | | | |
| **For the remainder of the questionnaire, please refer to the following definitions:**  **•** Regular partner refers to your boyfriend, partner, spouse, or any person whom you are in a long-term sexual relationship with.  • Casual partner refers to a person who is not paid for sex and whom you are not in a long-term sexual relationship with.  • Sex worker / Money Boy refers to a person whom you pay in exchange for sex. | | | | | | | | | | | | | | | | | | | |
| **In the last 3 months, how often did you use a condom when having:** | | **Always** | | **> half the time** | | | | **Half the time** | | | | **< half the time** | | | **Never used a condom** | | | | **N/A** |
|  | Oral sex with a regular partner? |  | |  | | | |  | | | |  | | |  | | | |  |
|  | Anal sex with a regular partner? |  | |  | | | |  | | | |  | | |  | | | |  |
|  | Oral sex with a casual partner? |  | |  | | | |  | | | |  | | |  | | | |  |
|  | Anal sex with a casual partner? |  | |  | | | |  | | | |  | | |  | | | |  |
|  | Oral sex with a sex worker/money boy? |  | |  | | | |  | | | |  | | |  | | | |  |
|  | Anal sex with a sex worker/money boy? |  | |  | | | |  | | | |  | | |  | | | |  |

| **HIV Pre-Exposure Prophylaxis and Post-Exposure Prophylaxis** | | | | | | | | | | | | |
| --- | --- | --- | --- | --- | --- | --- | --- | --- | --- | --- | --- | --- |
|  | Have you heard of HIV Pre-Exposure Prophylaxis (PrEP)? | Yes, I am currently on PrEP  Yes, I have taken it but no longer do so  Yes, I have heard of it but not taken it 🡪 skip to Q50  No, I have never heard of it 🡪 skip to Q51 | | | | | | | | | | |
|  | How long have you been/were you on PrEP? | Less than 3 months  Between 3 to 6 months  Between 6 to 12 months  For more than a year | | | | | | | | | | |
|  | **If you have taken PrEP**, did you take it daily or on-demand? | **Please select all that apply:**  Daily PrEP  On-Demand PrEP | | | | | | | | | | |
|  | **If you have taken PrEP**, where did you purchase the medication for PrEP? | **Please select all that apply:**  Government-Run Clinic (e.g. DSC Clinic)  Government-Run Hospital (e.g. General hospital)  GP Clinic  Online supplier  From friends who are currently on PrEP  From friends who are HIV-positive  From local importers of generic drugs  Overseas (please specify where): ______________  Others (please specify): ______________________ | | | | | | | | | | |
|  | **If you have taken PrEP**, where did you receive your follow-up care for PrEP? | **Please select all that apply:**  Government-Run Clinic (e.g. DSC Clinic)  Government-Run Hospital (e.g. General hospital)  GP Clinic  Overseas (please specify where): ______________  Others (please specify): ______________________  I was able to take PrEP without the guidance of a doctor | | | | | | | | | | |
|  | **If you have not taken PrEP,** would you consider taking PrEP? | Yes  No | | | | | | | | | | |
|  | Have you heard of HIV Post-Exposure Prophylaxis (PEP)? | Yes, I have taken it before  Yes, I have heard of it but not taken it 🡪 skip to Q53  No, I have never heard of it 🡪 skip to Q53 | | | | | | | | | | |
|  | **If you have taken PEP**, where did you receive the treatment for PEP? | **Please select all that apply:**  Government-Run Clinic (e.g. DSC Clinic)  Government-Run Hospital (e.g. General hospital)  GP Clinic  Online supplier  From friends who have been prescribed PrEP/PEP  From local importers of generic drugs  Overseas (please specify where): ______________  Others (please specify): ______________________ | | | | | | | | | | |
| **Substance Use** | | | | | | | | | | | | |
|  | Have you ever used any of the following drugs or substances ***during*** sex (i.e. chemsex, chill fun, cf etc.)?? | **Please select all that apply:**  Alcohol  Poppers  Heroin  Crystal Meth (‘Ice’/’Cream’)  Marijuana  Ketamine  Ecstasy  GHB / GBL  Erectile dysfunction medication – e.g. Viagra | | | | | | | | | | |
|  | **In the last 3 months, how often did you use the following substances during sex, or for the purpose of sex?** | **Always** | | **More than half the time** | | **Half the time** | | | **Less than half the time** | | | **Did not use it for sex** |
|  | Alcohol |  | |  | |  | | |  | | |  |
|  | Popper |  | |  | |  | | |  | | |  |
|  | Heroin |  | |  | |  | | |  | | |  |
|  | Crystal Meth (‘Ice’ / ‘Cream’) |  | |  | |  | | |  | | |  |
|  | Marijuana |  | |  | |  | | |  | | |  |
|  | Ketamine |  | |  | |  | | |  | | |  |
|  | Ecstasy |  | |  | |  | | |  | | |  |
|  | GHB / GBL |  | |  | |  | | |  | | |  |
|  | Erectile dysfunction medication – e.g. Viagra, Cialis, Black Ants |  | |  | |  | | |  | | |  |
|  | How often did you inject any of the above substances in the last 3 months? | Always  More than half the time  Half the time  Less than half the time  Never | | | | | | | | | | |
|  | How often did you use more than one type of substance at the same time or simultaneously (i.e. mixing drugs or a ‘cocktail’ of drugs) during sex in the last 3 months? | Always  More than half the time  Half the time  Less than half the time  Never | | | | | | | | | | |
|  | How often did these chemsex sessions include 3 persons or more (i.e. orgies or group sex) in the last 3 months? | Always  More than half the time  Half the time  Less than half the time  Never | | | | | | | | | | |
| **Stigma and Discrimination** | | | | | | | | | | | | |
| **Perceived Homosexual Stigma** | | **Strongly Agree** | **Agree** | | | | **Disagree** | | | **Strongly Disagree** | | |
|  | Many people believe that gay men have psychological problems. |  |  | | | |  | | |  | | |
|  | Many people do not see gay men as real men. |  |  | | | |  | | |  | | |
|  | Most families would be disappointed to have a gay son. |  |  | | | |  | | |  | | |
|  | Many people think that gay men have HIV and will die of AIDS. |  |  | | | |  | | |  | | |
|  | Many people do not accept same-sex male couples. |  |  | | | |  | | |  | | |
|  | Many people believe that gay men should not hug, hold hands, or kiss in public. |  |  | | | |  | | |  | | |
| **Internalized Homosexual Stigma** | | **Strongly Agree** | **Agree** | | | | **Disagree** | | | **Strongly Disagree** | | |
|  | I have tried to stop being attracted to men. |  |  | | | |  | | |  | | |
|  | If someone offered me the chance to be completely heterosexual, I would accept the chance. |  |  | | | |  | | |  | | |
|  | I wish I wasn’t attracted to men. |  |  | | | |  | | |  | | |
|  | I would like to get professional help in order to be less sexually attracted to men. |  |  | | | |  | | |  | | |
|  | I feel that being attracted to men is a shortcoming for me. |  |  | | | |  | | |  | | |
| **HIV Testing Self-Efficacy** | | | | | | | | | | | | |
| **How confident are you that you could do each of the following? (If you have never done some of these things, answer whether you think you could do them)** | | **Not confident at all** | | **Not very confident** | | **Somewhat confident** | | | **Very confident** | | | **Completely confident** |
|  | Getting tested for HIV at least once per year |  | |  | |  | | |  | | |  |
|  | Getting an HIV test after sex that you perceive as risky or unsafe |  | |  | |  | | |  | | |  |
|  | Asking my doctor for an HIV test |  | |  | |  | | |  | | |  |
|  | Discussing sexual risk events with a doctor or nurse |  | |  | |  | | |  | | |  |
|  | Getting emotional and social support from others for your HIV testing experience |  | |  | |  | | |  | | |  |
|  | Knowing how long to wait after a sexual risk event before getting tested for HIV |  | |  | |  | | |  | | |  |
|  | Knowing where to go to get tested for HIV |  | |  | |  | | |  | | |  |
|  | Making an appointment or scheduling a visit to the HIV testing clinic |  | |  | |  | | |  | | |  |
|  | Finding a healthcare provider and linking yourself to care if you test positive for HIV |  | |  | |  | | |  | | |  |
|  | Getting emotional and social support from others if you test positive for HIV |  | |  | |  | | |  | | |  |
| **HIV Testing Social Norms** | | | | | | | | | | | | |
| Below are a list of statements regarding HIV testing in your community. Please indicate the answer that best describes your answer to each statement. | | **Strongly Agree** | | | **Agree** | | | **Disagree** | | | **Strongly Disagree** | |
|  | Most people who want to get tested are afraid to get tested |  | | |  | | |  | | |  | |
|  | Most people who get tested do not want others to find out they were tested |  | | |  | | |  | | |  | |
|  | Most people who want to get tested will tell their partners they want to get tested |  | | |  | | |  | | |  | |
|  | Most people want to get tested for HIV |  | | |  | | |  | | |  | |
|  | Most people have been tested for HIV |  | | |  | | |  | | |  | |
|  | Most people get tested for HIV only if they are sick |  | | |  | | |  | | |  | |
|  | HIV treatment is effective |  | | |  | | |  | | |  | |
|  | HIV treatment is affordable |  | | |  | | |  | | |  | |
|  | HIV treatment is easily available in my community |  | | |  | | |  | | |  | |
