# Supplementary figures and images for: Effect of a Popular Web Drama Video Series on HIV and Other Sexually Transmitted Infection Testing Among Gay, Bisexual, and Other Men Who Have Sex With Men in Singapore: Community-Based, Pragmatic, Randomized Controlled Trial
Source: J Med Internet Res. 2022 May 6;24(5):e31401. doi: 10.2196/31401 (PMC9123545; doi:10.2196/31401)

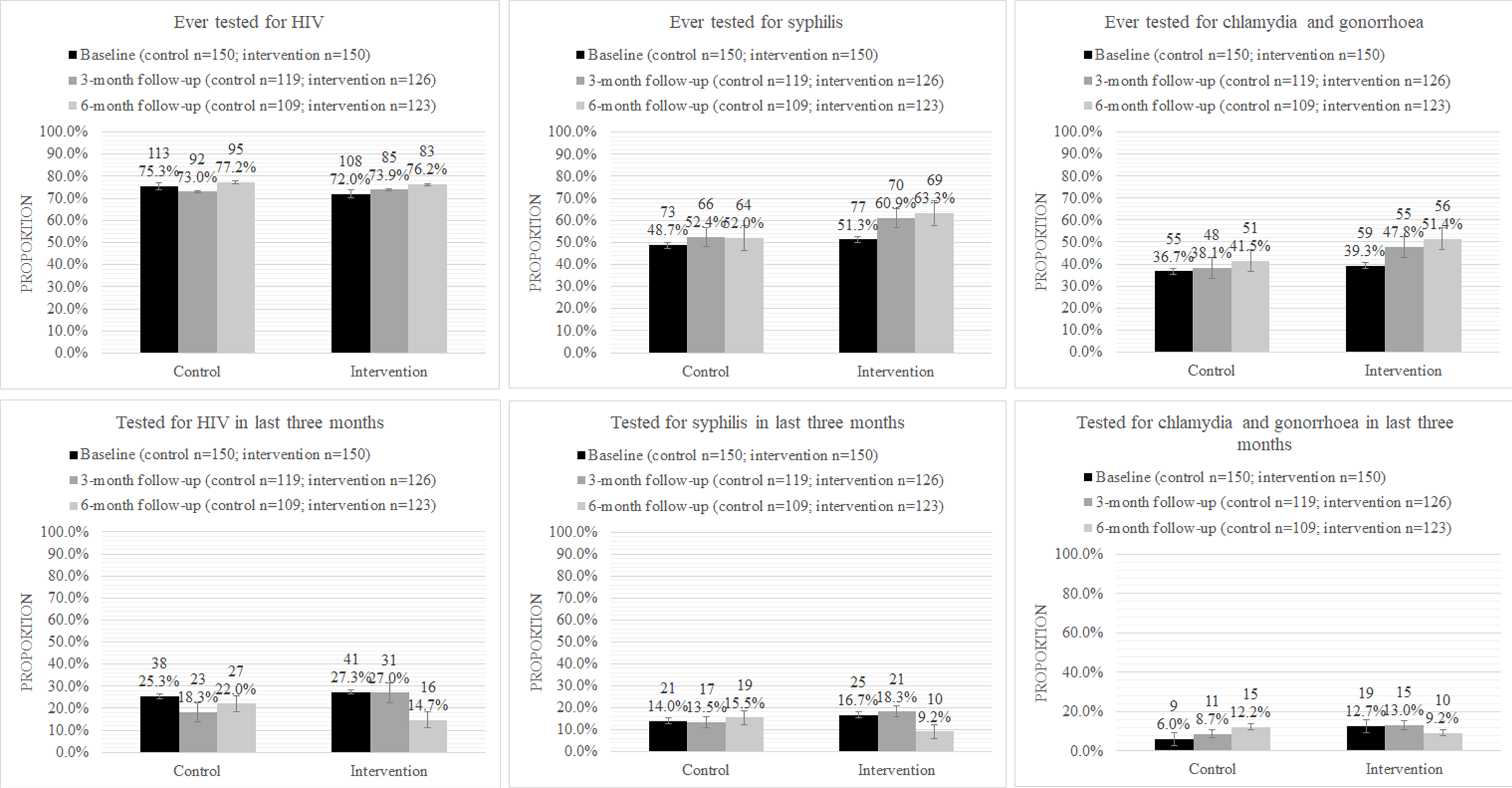

Supplement: Multimedia Appendix 2 [file jmir_v24i5e31401_app2.png]

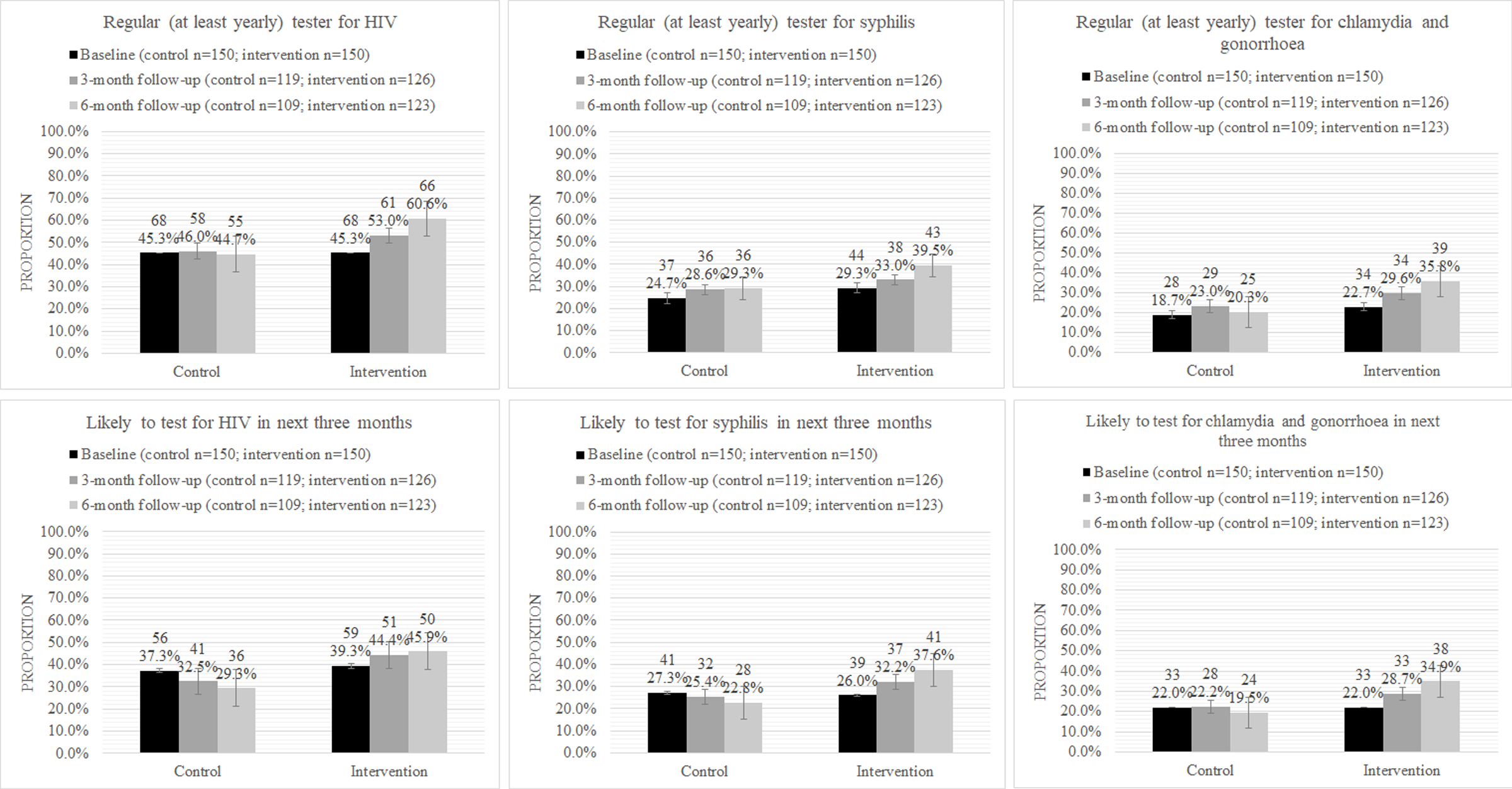

Supplement: Multimedia Appendix 3 [file jmir_v24i5e31401_app3.png]
