# Supplementary material for: Effect of a Popular Web Drama Video Series on HIV and Other Sexually Transmitted Infection Testing Among Gay, Bisexual, and Other Men Who Have Sex With Men in Singapore: Community-Based, Pragmatic, Randomized Controlled Trial
Source: J Med Internet Res. 2022 May 6;24(5):e31401. doi: 10.2196/31401 (PMC9123545; doi:10.2196/31401)
Supplement: Multimedia Appendix 4 [file jmir_v24i5e31401_app4.docx]

**Coronavirus Disease 2019 and its impact on the study**

The pragmatic nature of the trial also meant that the trial was conducted in a community-based setting, and subject to broader changes in the context in which it was rolled out. Recruitment for the trial began in October 2019, and recruitment of eligible participants for the baseline, randomization, and thus the allocation of the treatment took place up until end-December 2019. The next follow-up period at the 3-month time point then began from end-January 2020, from when the first survey was completed in the first follow-up, and lasted up until end-March 2020.

However, it was also during this time that the Coronavirus Disease 2019 (COVID-19) started to take its hold as a pandemic, with the first case reported in Singapore on 23 January 2020. Since COVID-19 was first reported in Singapore, the authorities have incrementally implemented a series of movement control measures to curb the spread of SARS-CoV-2, including the closure of entertainment establishments such as nightclubs and bars in late February 2020 following the change in Singapore’s Disease Outbreak Response System Condition (DORSCON) color code from yellow to orange, and a stricter set of ‘circuit-breaker’ measures in April 2020 that saw the closure of all but ‘essential’ workspaces in Singapore in response to the increasing rate of spread of COVID-19 in the community. While HIV and STI testing services are considered essential service, one of the test sites which is popular among GBMSM was closed briefly and subsequently resumed operations at reduced capacity. Furthermore, the circuit breaker period made it mandatory for individuals to stay at home and not visit individuals from other households, or risk imprisonment for up to six months, or a fine of up to SGD 10,000.00, or both under the COVID-19 (Temporary Measures) Act 2020.

**Figure S1** summarizes the timeline of events, including the phases of this trial study in the context of the events surrounding COVID-19 in Singapore. Overall, this would have had an impact on the primary outcomes of the trial in two specific ways. First, access to HIV and other STI testing services at the test site popular among GBMSM may have been limited during the periods when the first follow-up survey was disseminated, as well as the period after the first follow-up until the end of the trial in which the ‘circuit breaker’ movement control legislation was in full force. This would have led to a reduction in the overall levels of HIV and other STI testing in both arms. Second, as individuals were not allowed to meet others who did not belong to the same household, it is less likely that individuals would have engaged in behaviors that put them at a higher perceived risk for acquiring HIV and other STI, and thus would also mean a reduction in the overall levels of HIV and other STI testing. The results of the trial corroborate these statements.

Overall, we observed a decrease in recent testing for HIV and other STI among participants in both arms, as well as a concomitant decrease in perceived HIV and other STI risk among participants, likely due to a decrease in sexual activity among participants across both groups. We corroborate this with evidence presented in **Table S1** below, detailing the total number of unique sex partners that participants had in the previous three months at each time point of the trial. We also observed a drop in the rates of inconsistent condom use for anal sex with casual partners, as well as incidence of STI across both arms as well throughout the circuit breaker period.

**Figure S1.** Timeline of study in the context of COVID-19.


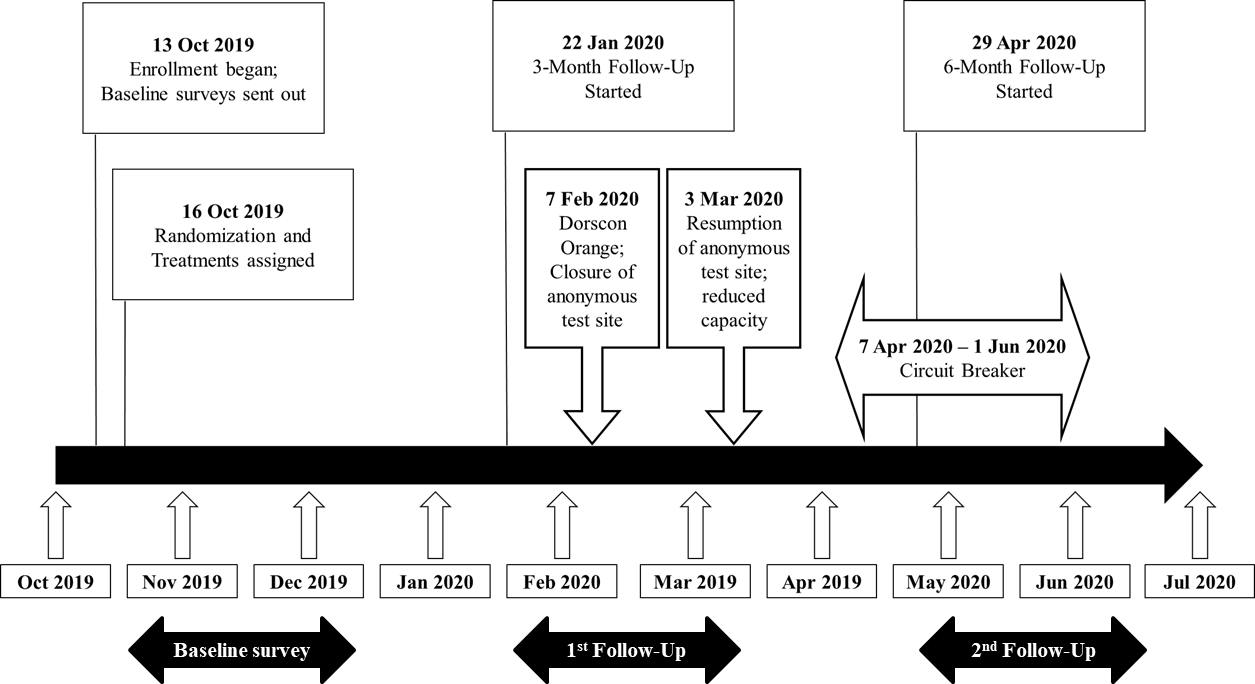


**Table S1.** Number of sexual partners in the last three months among participants across both study arms.

| **Number of sexual partners in last three months** | | **Control (n=150)** | | | | **Intervention (n=150)** | | | |  |
| --- | --- | --- | --- | --- | --- | --- | --- | --- | --- | --- |
|  |  | **Mean** | **SD** | **Median** | **IQR** | **Mean** | **SD** | **Median** | **IQR** |  |
| Baseline | | 2.7 | 4.28 | 1.0 | 2.0 | 2.4 | 3.10 | 1.0 | 2.0 |  |
| 3-Month Follow Up | | 2.7 | 3.84 | 1.0 | 2.0 | 2.1 | 2.56 | 1.0 | 2.0 |  |
| 6-Month Follow Up | | 1.7 | 3.00 | 1.0 | 2.0 | 1.6 | 1.50 | 1.0 | 1.0 |  |
|  |  |  |  |  |  |  |  |  |  |  |
| Abbreviation: SD, Standard Deviation; IQR, Inter-Quartile Range | | | | | | | | | | |
